# Medical Hints

**Published:** 1893-09

**Authors:** 


### Medical Hints.

BY C. H. M.

In the earlier days, perhaps the earlier years of your practice, you will have much leisure time. See to it that this time is well spent. Allow no opportunity for reading or study to pass. While reading alone will not make you a successful practitioner, it will add to your acquirements and you can never spend too much time in self-improvement. The reserve of knowledge that you gain will some day be of service to you.

Avoid the tendency to become onesided in your profession. Do not become so saturated with the odor of your drugs and medicines as to carry them into the drawing-room with you. Nothing is more offensive to well bred people than a boastful physician—one that seizes upon every opportunity to "talk shop" to his acquaintances. There is a certain unspoken etiquette, a certain air of good breeding that the public cannot fail to recognize and pay homage to if you but once possess it. Seek to cultivate an air of forgetfulness of your profession when thrown into contact with people socially.

Avoid medical controversies. They can lead to no good. You are not called upon to correct every rumor or statement that may come to your notice. There is an old adage that cannot be too carefully borne in mind: "Be silent when fools talk." No man can build up a reputation by his efforts to tear down another's. Sooner or later such men sink to their own level and they will be indelibly stamped with the mark of their real value.

One of the drawbacks to the advancement of scientific medicine to-day is the uneducated physician. A superficially educated man; a man who is contemptible enough to profit financially by the ignorance of the people who seek his counsel; a man dishonest enough to impose upon the credulity of patients; a man who pretends to know what he does not know; a man devoid of the ability to reason and reach conclusions. These are the men that to-day lower the standard of the medical profession and cause it to be regarded with distrust. Our medical colleges are beginning none too soon to demand more rigid qualifications than ever before.

---

Perseverance and determination are absolutely essential to success in the practice of medicine. Perhaps in no other profession will the powers of endurance and patience be so severely tested. The nature of your profession forbids your pushing yourself forward as many other professional men are permitted to do, and you will simply be compelled to wait until your services are sought. Though a modest way of showing your professional acquirements is not contrary to the "spirit" of your profession, yet anything that savors of boastfulness and impudence will at once be denounced.

Probably after you have gained a practice and a competence, after you have reached the point when you are frequently in demand and your professional opinion has become of worth to the profession and your patients, you will wonder how you acquired the position that once seemed so unattainable. Study the past—study your fellow men, learn if possible the source of your success and strive to improve upon it. In the practice of medicine, as elsewhere, always aim to be progressive, keep abreast of the times both in special and general literature. Every item of knowledge you acquire, every fact you learn, all serve to advance you to the front ranks and keep you there. Medicine is progressive. You cannot enter it and stand still. You must move ahead or fall to the rear.

